# Etiology and clinical characteristics of infantile cholestasis: a single-center retrospective study of 326 cases

**DOI:** 10.3389/fped.2026.1843435

**Published:** 2026-04-22

**Authors:** Yun-Ping Tang, Xu-Xia Wei, Ning Xue, Hai-Ying Yang, Hua Li

**Affiliations:** Department of Gastroenterology, Children's Hospital Affiliated to Shandong University (Jinan Children's Hospital), Jinan, Shandong, China

**Keywords:** bile acid profile, cholestasis, etiological analysis, genetic testing, infants

## Abstract

**Objective:**

To retrospectively investigate the clinical characteristics and etiological spectrum of infantile cholestasis, with an emphasis on evolving diagnostic approaches.

**Methods:**

Clinical data of 326 infants diagnosed with infantile cholestasis at the Children's Hospital Affiliated to Shandong University from January 2020 to December 2025 were retrospectively analyzed. Etiological distribution was systematically examined. Serum bile acid profiling was performed for suspected bile acid synthesis defects, and genetic sequencing for unexplained or suspected genetic cholestasis.

**Results:**

Among 326 infants with infantile cholestasis, 56.7% presented with light- or clay-colored stools, 62.9% had hepatomegaly, and 8.3% had comorbidities. The etiological spectrum included biliary tract anomalies [50.6%, including 161 biliary atresia (BA)], genetic metabolic liver diseases (9.8%, *n* = 32), infectious causes (7.4%), drug-related causes (3.4%), idiopathic cholestasis (6.7%), other rare causes (0.9%), and undetermined etiology (21.2%). No significant differences in sex or age were observed between the genetic metabolic group (*n* = 32) and BA group (*n* = 161) (both *P* > 0.05). After excluding 165 surgical cases, genetic testing was performed in 55 of 161 remaining infants (34.2%), with pathogenic or likely pathogenic variants identified in 33 (60.0% detection rate) across 14 genes (e.g., *JAG1*, *SLC25A13*, *ABCC2*). In an exploratory subgroup analysis (genetic metabolic, *n* = 16; BA, *n* = 20), the BA subgroup showed significantly higher levels of matrix metalloproteinase-7 (MMP-7), direct bilirubin, and GGT (*P* = 0.002 for GGT), with no other significant differences between the two subgroups.

**Conclusion:**

The etiology of infantile cholestasis is complex and highly heterogeneous. Genetic testing improves the diagnostic yield of inherited metabolic liver diseases. Serum bile acid profiling provides metabolomic signatures for etiological differentiation. Conventional liver function tests combined with serum MMP-7 represent a simple, reliable, noninvasive approach for early differentiation of biliary atresia.

## 1 Introduction

Infantile cholestasis, most prevalent within the first three months of life, refers to a spectrum of liver disorders characterized by conjugated hyperbilirubinemia resulting from impaired bile formation, secretion, and/or excretion due to intrahepatic or extrahepatic causes (1). Clinical manifestations are highly variable and include jaundice, pruritus, acholic stools, hepatomegaly or altered liver consistency, and malabsorption of fats and fat-soluble vitamins. These features frequently result in diarrhea, failure to thrive, and developmental delay, while severe cases may be accompanied by neuropsychiatric abnormalities (2).

Given its multifactorial etiology, infantile cholestasis remains a major cause of morbidity and mortality in early infancy (3). Recent advances in diagnostic modalities have revolutionized the characterization of its disease spectrum. In addition to classic etiologies such as biliary atresia (BA), parenteral nutrition-associated cholestasis, and infectious causes, the diagnostic yield for genetic and metabolic liver diseases has increased substantially. Early etiological diagnosis is pivotal for targeted intervention and favorable outcomes; nevertheless, atypical presentations in certain disorders create diagnostic dilemmas, increasing the risk of delayed or missed diagnosis and subsequent adverse clinical consequences.

Despite the availability of diagnostic guidelines, the etiological spectrum of infantile cholestasis varies across populations, and the differentiation between genetic metabolic liver disease and biliary atresia remains challenging. Therefore, the present study was conducted to address the following research questions in a large cohort of Chinese infants: (1) What is the distribution of etiologies, particularly the proportion of genetic metabolic liver diseases? (2) Can serum bile acid profiling and MMP-7 measurement reliably distinguish genetic metabolic liver disease from biliary atresia? (3) What is the diagnostic yield of genetic testing [including Whole-exome sequencing (WES)] in a selected subgroup of infants with unexplained or clinically suspected genetic cholestasis?

## 2 Materials and methods

### 2.1 Study design and study participants

A total of 326 infants diagnosed with infantile cholestasis at the Children's Hospital Affiliated to Shandong University between January 2020 and December 2025 were retrospectively enrolled. All consecutive eligible cases during this period were enrolled. The diagnosis of infantile cholestasis was established in accordance with the guidelines proposed by the North American Society for Pediatric Gastroenterology, Hepatology and Nutrition (NASPGHAN) (2): serum direct bilirubin >1.0 mg/dL in patients with total bilirubin <5 mg/dL, or direct bilirubin accounting for ≥20% of total bilirubin in patients with total bilirubin ≥5 mg/dL. This study was conducted in accordance with the Declaration of Helsinki and approved by the Ethics Committee of the Children's Hospital Affiliated to Shandong University (Approval No. SDFE-IBR/P-2023040).

### 2.2 Clinical data collection

Comprehensive clinical data were collected for all patients, including detailed medical history and physical examination findings. Laboratory assessments included liver function tests, total bile acids, lipid profile, blood ammonia, lactate, fasting blood glucose, alpha-fetoprotein, thyroid function, complete blood count, coagulation function, pathogen screening, inherited metabolic disease screening, routine urinalysis, mid-stream urine bacterial culture, and abdominal ultrasonography. For the subgroup analysis, selected patients underwent additional examinations, including serum matrix metalloproteinase-7 (MMP-7) measurement, serum bile acid profiling, and genetic testing. Patients were selected consecutively based on serum sample availability and enrollment order. Serum bile acid profiling was performed using liquid chromatography-tandem mass spectrometry (LC-MS/MS) for quantitative analysis of 20 bile acid species (detailed in the Appendix), as well as quantification of total cholic acid (Total-CA), total chenodeoxycholic acid (Total-CDCA), total ursodeoxycholic acid (Total-UDCA), total deoxycholic acid (Total-DCA), and total bile acids.

### 2.3 Diagnostic criteria

#### 2.3.1 Infectious diseases

Diagnoses were confirmed based on positive infectious markers. CMV infection was diagnosed based on positive CMV DNA detection in plasma or whole blood via quantitative PCR, combined with compatible clinical features and evidence of end-organ involvement, according to ECCI consensus (4). Urinary tract infection or sepsis was defined by at least two consecutive positive cultures and clinical improvement with appropriate antimicrobial treatment.

#### 2.3.2 Biliary tract abnormalities

##### 2.3.2.1 Biliary atresia (BA)

Diagnosed in accordance with international consensus criteria (5), based on clinical manifestations, biochemical profiles, imaging findings, surgical exploration, and liver histopathology.

##### 2.3.2.2 Choledochal cyst

Diagnosed based on imaging and intraoperative findings.

##### 2.3.2.3 Intrahepatic bile duct hypoplasia

Diagnosed by liver biopsy combined with genetic testing.

#### 2.3.3 Genetic and metabolic disorders

Diagnoses were confirmed by abnormal laboratory results, including blood glucose, thyroid function, blood/urine tandem mass spectrometry, serum cortisol, karyotype analysis, and genetic testing. Since MMP-7 is a component of the bile acid profiling assay, performing this test is generally advised in infants suspected of having biliary atresia or disorders of bile acid metabolism, and parental approval was required prior to testing. Genetic testing was performed only after biliary atresia had been excluded by ultrasonography and/or intraoperative cholangiography, or when clinical features suggested an inherited metabolic liver disease.

WES was used directly as the first-tier genetic test, without an initial targeted gene panel. Patients were eligible for WES if they met any of the following criteria: (1) Unexplained etiology after a complete initial workup [including liver function tests, gamma-glutamyl transferase (GGT), bile acid profiling, abdominal ultrasound, and CMV screening]; (2) High clinical suspicion of a genetic disorder (e.g., Alagille syndrome, progressive familial intrahepatic cholestasis); (3) Family history of cholestasis or consanguineous marriage; (4) Low-GGT cholestasis with no significant abnormalities on bile acid profiling; (5) Recurrent or persistently unexplained cholestasis; (6) Parental request or preference for genetic testing.

Pathogenicity of genetic variants was assessed according to the ACMG/AMP 2015 guidelines (6). Standard criteria were applied, including population frequency (PM2), computational prediction data (PP3), functional studies (PS3), segregation analysis (PP1), and variant type (PVS1). Variants were classified as pathogenic, likely pathogenic, variant of uncertain significance (VUS), likely benign, or benign. Only pathogenic and likely pathogenic variants were reported as diagnostic findings. All identified variants were manually reviewed by two independent investigators to ensure consistency.

#### 2.3.4 Parenteral nutrition-associated cholestasis

Cholestasis occurring after ≥14 consecutive days of total parenteral nutrition, with other causes excluded.

#### 2.3.5 Idiopathic infantile cholestasis

No definite etiology identified after comprehensive diagnostic evaluation.

### 2.4 Study grouping

**Figure 1:**
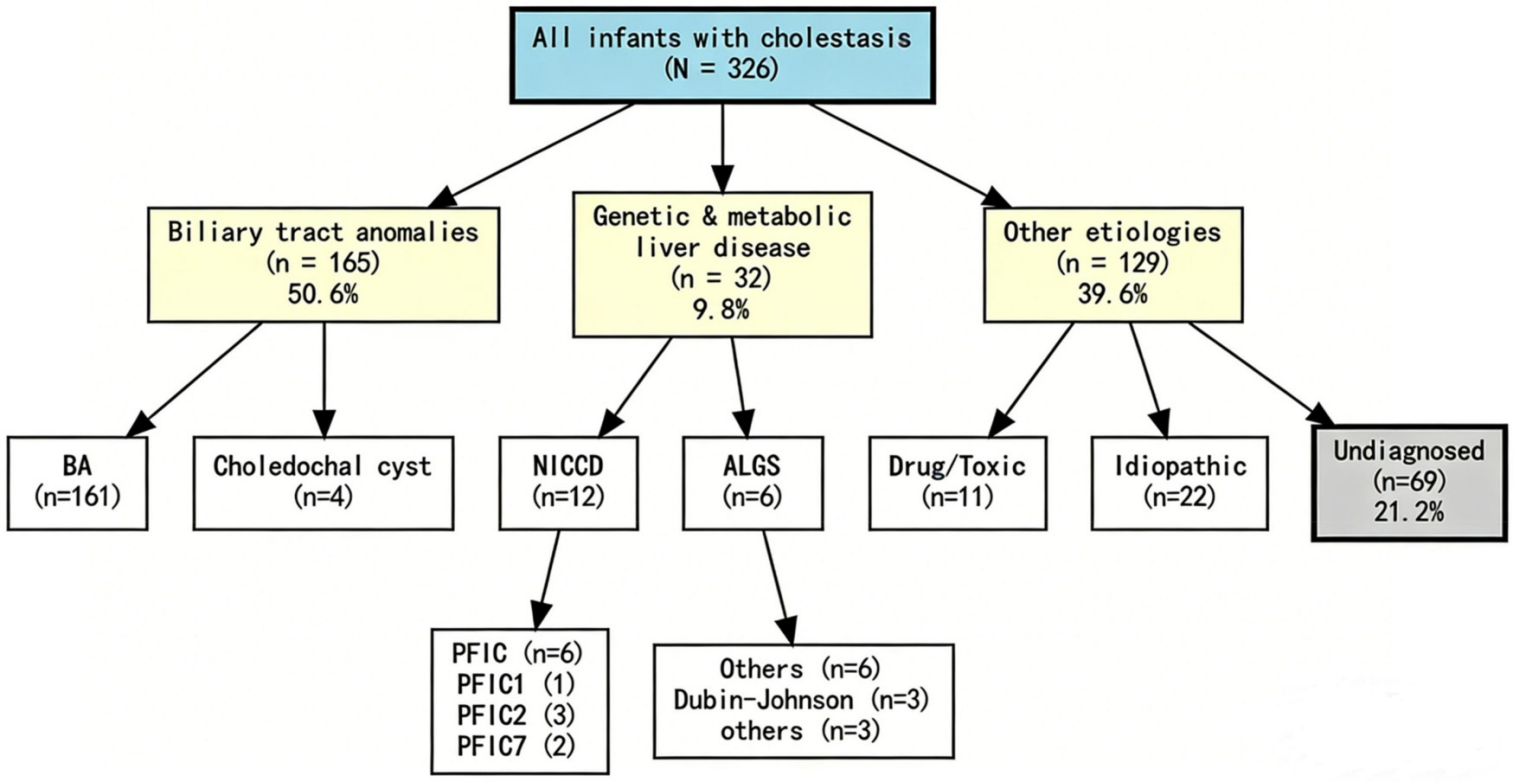
Etiological spectrum of infantile cholestasis in the study cohort. A total of 326 infants with cholestasis were enrolled. The cohort was categorized into three main etiological groups: biliary tract anomalies (*n* = 165, 50.6%), genetic and metabolic liver disease (*n* = 32, 9.8%), and other etiologies (*n* = 129, 39.6%). The “other etiologies” group included infectious causes (*n* = 24, 7.4%), drug/toxic factors (*n* = 11, 3.4%), idiopathic infantile cholestasis (*n* = 22, 6.7%), other rare causes (*n* = 3, 0.9%), and undiagnosed cases (*n* = 69, 21.2%). Detailed subgroup classifications are shown within each category.

Patients were categorized into two groups for comparative analysis: the genetic and metabolic liver disease group (Group GMLD, *n* = 32) and the biliary atresia group (Group BA, *n* = 161). A subgroup analysis including serum MMP-7 measurement and bile acid profiling was performed in a subset of patients (GMLD subgroup, *n* = 16; BA subgroup, *n* = 20). The etiological spectrum and diagnostic features of infantile cholestasis were systematically analyzed. A schematic diagram illustrating the etiological classification of infantile cholestasis is provided in Figure 1 to enhance readability.

### 2.5 Statistical analysis

To minimize selection bias, all consecutive eligible cases were enrolled. Data were collected using a standardized form and independently extracted by two researchers to reduce information bias. Missing data were <5% for all variables; therefore, complete-case analysis was used without imputation. Due to the retrospective design, no prospective power calculation was performed. A *post-hoc* analysis showed adequate overall power (>80%) for the primary analysis. For the GMLD (*n* = 32) vs. BA (*n* = 161) comparison, the small GMLD group precluded formal matching or adjustment (e.g., propensity score weighting), as such methods would yield unstable estimates or significant data loss; these results are therefore exploratory and should be interpreted with caution. For the GMLD subgroup (*n* = 16) vs. BA subgroup (*n* = 20), *post-hoc* power was 85%–90% for MMP-7 (*P* < 0.001) and 80%–85% for GGT (*P* = 0.002) (two-sided alpha = 0.05), indicating adequate power to detect the primary positive findings. All power calculations were performed using GPower version 3.1.

Statistical analyses were performed using SPSS 27.0 software (IBM Corp., Armonk, NY, USA). Categorical variables were presented as case numbers and percentages (n, %). Normally distributed continuous variables were expressed as mean ± standard deviation (SD), and non-normally distributed variables as median (interquartile range, IQR). Between-group comparisons were performed using the chi-square test, independent-samples t-test, or Mann–Whitney U test as appropriate. A *P*-value < 0.05 was considered statistically significant.

## 3 Results

### 3.1 Etiological spectrum of infantile cholestasis

All 326 infants presented with varying degrees of jaundice. The cohort comprised 184 males (56.4%) and 142 females (43.6%), with a male-to-female ratio of approximately 1.3:1. The median age at admission was 60 days (range 1 day to 1 year). Light- or clay-colored stools were observed in 185 patients (56.7%), and 205 patients (62.9%) had hepatomegaly with or without splenomegaly. Twenty-seven patients (8.3%) had comorbidities, including congenital heart disease, umbilical hernia, hearing impairment, developmental delay, and hemangioma. The etiological distribution of the 326 infants is shown in Figure 1.

The etiological distribution was summarized as follows. Infectious factors accounted for 24 cases (7.4%), comprising CMV infection (*n* = 22) and sepsis (*n* = 2). Biliary tract abnormalities were identified in 165 cases (50.6%), including BA (*n* = 161) and choledochal cysts (*n* = 4). As described in Study Grouping, the GMLD group (*n* = 32) and BA group (*n* = 161) were selected for subsequent comparative analysis. Genetic metabolic liver diseases were diagnosed in 32 patients (9.8%), encompassing neonatal intrahepatic cholestasis caused by citrin deficiency (NICCD, *n* = 12), Alagille syndrome (ALGS, *n* = 6), progressive familial intrahepatic cholestasis (PFIC, *n* = 6), and Dubin-Johnson syndrome (*n* = 3). Additional rare genetic disorders were identified in 5 patients, including immunodeficiency type 70, primary carnitine deficiency, *HYDIN*-related disorder, *FOXN1* deficiency, and hepatic veno-occlusive disease with immunodeficiency. Among the PFIC cases, pathogenic variants were identified in *ATP8B1* (PFIC1, *n* = 1), *ABCB11* (PFIC2, *n* = 3), and *USP53* (PFIC7, *n* = 2). Drug/toxic factors, primarily parenteral nutrition-associated cholestasis, were observed in 11 cases (3.4%). Idiopathic infantile cholestasis was diagnosed in 22 cases (6.7%). Other rare causes accounted for 3 cases (0.9%): one case each of Kawasaki disease, autoimmune hemolytic anemia, and pituitary stalk interruption syndrome. Additionally, 69 patients (21.2%) remained without a definitive etiological diagnosis due to incomplete diagnostic workup.

### 3.2 Comparison between genetic metabolic liver disease and BA groups

Consistent with the Study Grouping section, the genetic metabolic liver disease group (Group GMLD, *n* = 32) included 14 males and 18 females, with a median age at admission of 2.6 months (range 1.0 month to 12.0 months). The BA group (Group BA, *n* = 161) consisted of 84 males and 77 females, with a median age of 1.6 months (range: 0.03 to 12 months). There were no significant differences between the two groups in sex distribution (*χ*^2^ test, *P* > 0.05) or age at presentation (Mann–Whitney U test, *P* > 0.05). However, given the substantial imbalance in sample sizes (32 vs. 161), these null findings should be interpreted with caution due to limited statistical power.

As predefined in the subgroup analysis within Study Grouping, serum bile acid profiling and serum MMP-7 measurement was performed in a subset of patients (GMLD subgroup, *n* = 16; BA subgroup, *n* = 20). Notably, serum MMP-7 concentrations were significantly higher in the BA subgroup than in the GMLD subgroup (U = 51, *P* < 0.001; Table 1). Analysis of liver function parameters showed that direct bilirubin and GGT levels were significantly higher in the BA subgroup (*P* = 0.002 for GGT; Table 1), whereas no other significant differences were observed between the two subgroups. Conversely, the GMLD subgroup exhibited significantly higher concentrations of conjugated bile acids including glycocholic acid (GCA), glycochenodeoxycholic acid (GCDCA), glycoursodeoxycholic acid (GUDCA), glycohyodeoxycholic acid (GHDCA), taurocholic acid (TCA), taurochenodeoxycholic acid (TCDCA), and tauroursodeoxycholic acid (TUDCA) as well as higher total bile acid fractions (Total-CA, Total-CDCA, Total-UDCA, and total bile acids) compared with the BA subgroup (*P* < 0.05). There were no significant between-subgroup differences in free bile acid levels. Detailed data are shown in Table 2.

**Table 1:** Comparison of parameters between GMLD subgroup (*n* = 16) and BA subgroup (*n* = 20).

Parameter	GMLD Subgroup (*n* = 16) Median (IQR)	BA Subgroup (*n* = 20) Median (IQR)	*P* value
MMP-7 (ng/mL)	11.99 (4.38–76.89)	54.24 (7.88–130.20)	**<0**.**001**
ALT (U/L)	104.5 (47.8–256.5)	152.0 (70.8–190.5)	0.496
AST (U/L)	149.0 (76.5–363.5)	196.0 (133.3–316.0)	0.423
TBIL (*μ*mol/L)	157.4 (120.1–190.3)	185.6 (154.3–209.4)	0.056
DBIL (μmol/L)	97.9 (68.4–139.3)	118.2 (88.1–140.4)	**0**.**018**
GGT (U/L)	88.0 (51.3–155.5)	261.0 (118.5–490.5)	**0**.**002**
TBA (μmol/L)	158.2 (125.9–263.8)	125.6 (85.1–166.6)	0.099

Data are presented as median (interquartile range, IQR) due to non-normal distribution. *P* values were calculated using the Mann-Whitney U test. Statistically significant differences (*P* < 0.05) are shown in bold.

GMLD, genetic and metabolic liver disease; BA, biliary atresia; MMP-7, matrix metalloproteinase-7; ALT, alanine aminotransferase; AST, aspartate aminotransferase; TBIL, total bilirubin; DBIL, direct bilirubin; GGT, gamma-glutamyl transferase; TBA, total bile acids.

**Table 2:** Comparison of serum bile acid profiles between GMLD subgroup (*n* = 16) and BA subgroup (*n* = 20).

Variable	Group A Median (Q1, Q3)	Group B Median (Q1, Q3)	Z	*P*
CA	0.04 (0.04, 0.04)	0.04 (0.04, 0.04)	-0.936	0.349
CDCA	0.04 (0.03, 0.05)	0.03 (0.03, 0.04)	-1.248	0.212
UDCA	0.04 (0.04, 0.05)	0.04 (0.04, 0.04)	-1.892	0.059
GCA	12.45 (6.02, 31.85)	8.96 (4.14, 14.70)	-2.021	0.043
GCDCA	43.30 (20.98, 61.86)	26.65 (16.22, 40.18)	-2.312	0.021
GUDCA	0.21 (0.06, 15.64)	0.76 (0.05, 8.32)	-2.064	0.039
GHDCA	0.04 (0.04, 0.04)	0.04 (0.04, 0.05)	-1.978	0.048
TCA	20.23 (14.14, 29.72)	18.58 (11.40, 27.38)	-2.384	0.017
TCDCA	42.92 (27.01, 66.16)	34.59 (22.12, 50.90)	-2.541	0.011
TUDCA	0.29 (0.05, 8.11)	0.10 (0.03, 7.75)	-2.171	0.030
Total-CA	37.43 (23.11, 69.79)	24.42 (18.01, 40.14)	-2.684	0.007
Total-CDCA	83.86 (43.74, 168.66)	56.32 (27.30, 80.69)	-2.502	0.012
Total-UDCA	0.51 (0.11, 28.85)	0.82 (0.15, 27.30)	-2.045	0.041
Total-BA	173.67 (101.61, 240.20)	110.33 (76.00, 142.09)	-2.348	0.019

Data are presented as median (Q1, Q3). Due to skewed distribution, between-group comparisons were performed using the Mann-Whitney U test. Statistically significant differences (*P* < 0.05) are shown in bold. These findings should be interpreted with caution given the skewed data distribution and small sample sizes.

GMLD, genetic and metabolic liver disease; BA, biliary atresia; CA, cholic acid; CDCA, chenodeoxycholic acid; UDCA, ursodeoxycholic acid; GCA, glycocholic acid; GCDCA, glycochenodeoxycholic acid; GUDCA, glycoursodeoxycholic acid; GHDCA, glycohyodeoxycholic acid; TCA, taurocholic acid; TCDCA, taurochenodeoxycholic acid; TUDCA, tauroursodeoxycholic acid; Total_CA, total cholic acid; Total_CDCA, total chenodeoxycholic acid; Total_UDCA, total ursodeoxycholic acid; Total_Bile_Acids, total bile acids.

### 3.3 Genetic analysis of major genetic metabolic liver diseases

After excluding 165 cases of biliary tract anomalies (161 biliary atresia and 4 choledochal cysts) for which genetic testing is not routinely indicated, the remaining 161 infants with cholestasis of suspected genetic or unknown etiology underwent analysis. Of these, 55 underwent genetic testing (testing rate 34.2%), and 33 were found to carry pathogenic or likely pathogenic variants (genetic detection rate 60.0%). A total of 33 pathogenic variants were identified in 14 distinct genes associated with genetic metabolic liver diseases, including *JAG1*, *SLC25A13*, *ABCC2*, *ATP8B1*, *ABCB11*, *USP53*, *IVNS1ABP*, *SLC22A5*, *HYDIN*, *FOXN1*, and *SP110*. Detailed information on the mutated genes, inheritance patterns, and parental origins of the mutations is summarized in Table 3.

**Table 3:** Genetic variants identified in patients with genetic metabolic liver diseases.

Disease	n	Gene	Inheritance Pattern	Maternal Mutation/Paternal Mutation
Alagille syndrome (ALGS)	4	JAG1	AD	c.3,385C > A (p.H1,129N) M/– P; c.954_955dup (p. Lys319ThrfsTer94) M/– P; c.1,452_1,453del (p.C484fs) *de novo*; –M/ c.2,578_2,582delinsTGATTCC (p. Gly860Ter) P
	2	20p12.3p12.2	AD (*de novo*)	3.79 Mb deletion (includes *JAG1*)—M/– P (proband only)
Neonatal intrahepatic cholestasis caused by citrin deficiency (NICCD)	8	SLC25A13	AR	c.1,638_1,660dup (p.Ala554Glyfs17) M/IVS16ins3 kb splicing P; c.852_855delTATG (p.Met285Phefs2) M/c.1,078C > T (p.Arg360*) P; c.1,638_1,660dup (p.Ala554Glyfs17) M/IVS4ins6 kb splicing P; c.852_855delTATG (p.Met285Phefs2) M/c.1,638_1660dup (p.Ala554Glyfs17) P; c.852_855delTATG (p.Met285Phefs2) M/c.852_855delTATG (p.Met285Phefs2) P; c.775C > T (p.Gln259) M/c.1,078C > T (p.Arg360*) P; c.1,081G > A (p.Ala340Thr) M/c.852_855del (p.Arg284fs) P; c.1,754G > A (p.Arg585His) M/c.615 + 5G > A (splicing) P
	4	SLC25A13	AR	c.852_855delTATG (p.M285pfs*2) M/c.852_855delTATG (p.M285pfs*2) P
Dubin-Johnson syndrome	2	ABCC2	AR	c.2,026G > C (p. Gly676Arg) M/c.3,825C > G (p. Tyr1,275Ter) P; c.1,530G > C (p.K510N) M/c.3,928C > T (p.R1,310X) P
Progressive familial intrahepatic cholestasis 1 (PFIC1)	1	ATP8B1	AR	c.2,965A > G (p. Asn989Asp) M/p. Arg941Ter P
Progressive familial intrahepatic cholestasis 2 (PFIC2)	3	ABCB11	AR	c.1,197 + 1G > T (splicing) M/c.382C > T (p.R128C) P; c.145C > T (p.Q49X) M/c.2,631delG (p.M878X) P; c.1,434 + 5G > A (p.?) M/c.2,782C > T (p. Arg928Ter) P
Progressive familial intrahepatic cholestasis 7 (PFIC7)	2	USP53	AR	c.1,426C > T (p. R476X) M/c.9G > A (p.W3X) P; c.1,024C > T (p. Gln342Ter) M/c.1,558C > T (p. Arg520Ter) P
Immunodeficiency type 70	1	IVNS1ABP	AD	– M/c.1,731A > C (p. Glu577Asp) P
Primary carnitine deficiency	1	SLC22A5	AR	c.808T > C (p. Cys270Arg) M/c.760C > T (p. Arg254Ter) P
HYDIN-related disorder	1	HYDIN	AR	c.3,979G > A M/– P
FOXN1 deficiency	1	FOXN1	AR	Not available
Hepatic veno-occlusive disease with immunodeficiency	1	SP110	AR	c.1,342C > T (p. Arg448Ter) M/c.193G > T (p. Val165Leu) P

–, mutation not identified; AD, autosomal dominant; AR, autosomal recessive; M, maternal origin; P, paternal origin.

Patients with 20p12.3p12.2 deletion (ALGS) are listed separately due to copy number variation (*de novo*).

Detailed mutation information was not available for FOXN1 deficiency.

## 4 Discussion

Infantile cholestasis encompasses a heterogeneous group of disorders with diverse etiologies, broadly classified into five major categories: obstructive, genetic metabolic, infectious, drug/toxic, and idiopathic. Given that therapeutic strategies and clinical outcomes vary considerably according to the underlying etiology, early and accurate diagnosis is critical for optimizing treatment and improving prognosis. A 2014 single-center study from Germany reported BA as the most common diagnosis (41%), followed by idiopathic cholestasis (13%), PFIC (10%), and prematurity-associated cholestasis (10%). ALGS, *α*₁-antitrypsin deficiency, and mitochondrial disorders each accounted for 2% of cases (7).

Consistent with previous reports, serum MMP-7 levels were significantly higher in Group BA than in Group GMLD (as defined in the Study Grouping section). In the same subgroup of patients (GMLD subgroup, *n* = 16; BA subgroup, *n* = 20), the median MMP-7 concentration was 54.24 ng/mL (range 7.88–130.20) in the BA subgroup and 11.99 ng/mL (4.38–76.89) in the GMLD subgroup**,** with mean values of 61.23 ± 35.12 ng/mL and 22.62 ± 23.65 ng/mL, respectively. The Mann–Whitney U test revealed a highly significant difference (U = 51, *P* < 0.001). These findings support that MMP-7 is a sensitive and specific noninvasive biomarker for the early differential diagnosis of BA, aligning with previous studies (8, 9).

Bile acids, the primary end products of cholesterol metabolism, are amphipathic molecules that promote intestinal fat absorption and act as critical signaling regulators of glucose, lipid, and energy homeostasis (10). Under physiological conditions, conjugation with taurine or glycine increases bile acid hydrophilicity, thereby reducing cytotoxicity and preventing hepatocellular injury (11). This compensatory mechanism is particularly relevant in cholestatic disorders, where impaired enterohepatic circulation leads to accumulation of potentially toxic bile acid species. Elevated levels of hydrophobic bile acids **-**including GCDCA, CA, and TCDCA **-**can compromise hepatocyte membrane integrity, trigger biliary epithelial injury, and ultimately induce hepatocyte apoptosis or necrosis (12, 13).

Consistent with these pathophysiological mechanisms, our study revealed distinct bile acid profiling patterns between etiological subgroups. In the same subgroup (GMLD subgroup, *n* = 16; BA subgroup, *n* = 20), patients in the GMLD subgroup exhibited significantly higher levels of conjugated bile acids**,** including GCA, GCDCA, GUDCA, GHDCA, TCA, TCDCA, and TUDCA**,** compared to those in the BA subgroup. These findings align with previous observations in NICCD, where defects in canalicular membrane transporters result in impaired bile acid excretion and subsequent elevation of conjugated bile acid species (14). The distinct metabolic signatures associated with different etiologies underscore the potential utility of serum bile acid profiling as a complementary diagnostic tool. However, as shown in Table 2, several bile acid indices**,** including GUDCA and TUDCA**,** exhibited markedly skewed distributions. Therefore, these findings should be interpreted with caution, and the clinical utility of individual bile acid species requires validation in larger prospective cohorts.

PFIC constitutes a group of autosomal recessive disorders characterized by impaired bile acid transport (15, 16). As a rare disease, PFIC has an estimated global incidence of 1 in 50,000 to 100,000 individuals (17). Among the 32 patients in the GMLD group, pathogenic mutations were identified in *ABCB11* (*n* = 3), *USP53* (*n* = 2), and *ATP8B1* (*n* = 1). *USP53* encodes ubiquitin-specific peptidase 53, a tight junction-associated protein involved in transmembrane transport, membrane permeability, apoptosis, and transcriptional regulation (18). Variants in *USP53* cause impaired bile acid excretion. Consistent with the bile acid profiling findings in the GMLD subgroup, serum bile acid analysis of the two affected children revealed markedly elevated levels of conjugated bile acids, including GCA, GCDCA, TCA, and TCDCA. This disruption leads to hepatocyte and biliary tract injury. The expanding identification of these genetic entities underscores the increasingly important role of advanced molecular diagnostics in clarifying previously unexplained cases of infantile cholestasis.

The evolving etiological landscape of infantile cholestasis reflected in our findings is partly attributable to advances in genetic testing technologies, which have substantially increased the diagnostic yield for inherited metabolic liver diseases. As illustrated in Figure 1, serum bile acid profiling offers metabolomic insights that may aid in etiological differentiation, while conventional liver function tests combined with MMP-7 levels provide simple, practical biomarkers for the early distinction of Group BA from Group GMLD.

Collectively, these findings highlight that advanced genetic testing, serum bile acid profiling, and MMP-7 measurement are valuable tools for improving etiological diagnosis and differentiation in infantile cholestasis. Nevertheless, as noted in the Results section, 21.2% of infants with cholestasis in this cohort remained without a definitive etiological diagnosis. This relatively high proportion warrants careful consideration. Several factors may account for this high proportion. First, as a retrospective study, some patients did not undergo a complete diagnostic workup due to limited clinical resources or loss to follow-up. Second, advanced diagnostic tools such as genetic sequencing and bile acid profiling were not universally performed in all patients but were reserved for those with specific clinical indications (as described in the GMLD and BA subgroup definitions), which may have led to underdiagnosis of certain metabolic or genetic disorders. Third, some patients with transient or self-limited cholestasis may have resolved before a definitive diagnosis could be established. Fourth, parental refusal of further testing also contributed to incomplete diagnostic ascertainment.

The high proportion of undetermined cases has important implications for the interpretation of our results. It is possible that some patients in the undetermined group actually had undiagnosed genetic metabolic liver diseases, which may have led to underestimation of the true GMLD proportion (9.8%) reported in this study. Furthermore, if a subset of undetermined cases shares biological or biochemical features with either Group GMLD or Group BA, the comparative analyses between these two groups might be biased. To address this limitation, future prospective studies should implement standardized diagnostic algorithms that include early genetic testing (e.g., whole-exome sequencing as a first-tier test) and comprehensive metabolic screening for all infants with unexplained cholestasis.

Several limitations of this study warrant consideration. First, the retrospective, single-center design at a tertiary referral hospital may have introduced selection bias, as our cohort likely overrepresents infants with severe or complex cholestasis while underrepresenting those with mild or self-limited disease managed in primary care, thereby limiting generalizability. Additionally, the marked sample size imbalance between the GMLD (*n* = 32) and BA (*n* = 161) groups precluded formal statistical adjustment, potentially introducing confounding bias. Consequently, the comparative findings between these two groups are exploratory and require validation in larger, well-balanced cohorts. Second, the 21.2% undetermined etiology rate represents a major limitation. Third, bile acid metabolism is influenced by multiple confounding factors that were not fully controlled, such as postnatal age, nutritional status (e.g., parenteral vs. enteral feeding), and residual liver function. Therefore, the observed differences between the GMLD (*n* = 16) and BA (*n* = 20) subgroups require validation in larger prospective cohorts. Fourth, no prospective sample size estimation was performed for the subgroup analyses due to the rarity of both conditions and the single-center design. These subgroup results should therefore be interpreted as exploratory with limited statistical power; nevertheless, we provide point estimates with 95% confidence intervals to serve as reference ranges for effect sizes. Fifth, genetic testing was not performed on all 326 infants. Patients with confirmed biliary tract anomalies (*n* = 165) were reasonably excluded, as genetic testing is not routinely indicated for these conditions in clinical practice. Thus, the reported testing and detection rates reflect a selected subgroup rather than the entire cohort. These findings underscore the need for standardized diagnostic algorithms and broader application of advanced genomic and metabolomic technologies.

Future multicenter prospective studies with larger sample sizes and standardized diagnostic protocols are warranted to validate our findings, refine etiological classification, and develop evidence-based diagnostic and therapeutic strategies for this complex and heterogeneous group of disorders.

## Data Availability

The datasets presented in this study can be found in online repositories. The names of the repository/repositories and accession number(s) can be found in the article/supplementary material.
